# The Antioxidant and In Vitro Wound Healing Activity of *Cotyledon orbiculata* Aqueous Extract and the Synthesized Biogenic Silver Nanoparticles

**DOI:** 10.3390/ijms232416094

**Published:** 2022-12-17

**Authors:** Caroline Tyavambiza, Mervin Meyer, Adedoja Dorcas Wusu, Abram Madimabe Madiehe, Samantha Meyer

**Affiliations:** 1 Department of Biomedical Sciences, Cape Peninsula University of Technology, P.O. Box 1906, Bellville 7535, South Africa; 2 DSI/Mintek Nanotechnology Innovation Centre, Department of Biotechnology, University of the Western Cape, Private Bag X17, Bellville 7530, South Africa; 3 Nanobiotechnology Research Group, Department of Biotechnology, University of the Western Cape, Private Bag X17, Bellville 7530, South Africa

**Keywords:** silver nanoparticles, wound healing, gene expression, cell proliferation, cell migration

## Abstract

The synthesis of silver nanoparticles using biogenic methods, particularly plants, has led to the discovery of several effective nanoparticles. In many instances, plant-derived silver nanoparticles have been shown to have more activity than the plant extract which was used to synthesize the nanoparticles. Silver nanoparticles have been successfully synthesized using the medicinal plant, *Cotyledon orbiculata.* This is a shrub found in the Western Cape province of South Africa. It has a long history of use in traditional medicine in the treatment of wounds and skin infections. The *C. orbiculata* synthesized silver nanoparticles (*Cotyledon*-AgNPs) were reported to have good antimicrobial and anti-inflammatory activities; however, their wound-healing properties have not been determined. This study aimed to determine the wound healing activity of *Cotyledon*-AgNPs using the scratch assay. Gene expression studies were also done to determine the nanoparticles’ mechanism of action. The *Cotyledon*-AgNPs showed good antioxidant, growth-promoting and cell migration properties. Gene expression studies showed that the *C. orbiculata* water extract and *Cotyledon*-AgNPs promoted wound healing by upregulating genes involved in cell proliferation, migration and growth while downregulating pro-inflammatory genes. This confirms, for the first time that a water extract of *C. orbiculata* and silver nanoparticles synthesized from this extract are good wound-healing agents.

## 1. Introduction

Green nanotechnology is receiving much attention in all scientific research areas worldwide. Its popularity can be attributed to the ability to synthesize biocompatible nanomaterials using simple, eco-friendly, and cost-effective methods. This field of nanotechnology involves the use of biological materials such as plants and microorganisms in nanomaterial synthesis. This significantly reduces energy costs, the use of toxic chemicals and some expensive materials and instruments which are common with the chemical and physical methods [1]. Recently, green nanotechnology has been applied in the form of nanoparticles, nanocomposites, hydrogels and nanofibers for the development of products that are used for wound treatment [2]. Metallic nanoparticles containing silver, gold and zinc are the most studied due to their unique properties most notably their antibacterial activity [3]. In addition to its antibacterial activity, silver promotes wound healing and has been used in wound dressings for the treatment of different types of wounds including burns and wounds [4]. Biogenic silver nanoparticles (AgNPs) have become popular antimicrobial, anti-inflammatory, and good wound healing agents [5]. The wound-healing potential of biogenic nanoparticles in both in vitro and in vivo models has been reported. 

South Africa is a richly biodiverse country with a strong history of traditional medicinal practice. It hosts around 30,000 flowering plant species and accounts for almost 10% of the world’s higher plant species [6,7]. *C. orbiculata* is one example of a plant that is used in traditional medicine in South Africa. It is used in home gardens as an ornamental plant, but its leaves are also sold as herbal medicines in the informal herbal medicine markets in different provinces of South Africa [8]. *C. orbiculata* commonly known as pig’s ear is a succulent shrub widely distributed in the Western Cape province of South Africa. It has a long history of use in traditional medicine. *C. orbiculata* has been used for the treatment of skin rashes, abscesses, sores and wounds, inflammation, boils, acne, corns and warts, earache, toothache, epilepsy and syphilis [9,10]. Studies have reported that *C. orbiculata* possesses various biological activities such as antibacterial, antifungal, anticonvulsant, antinociceptive, anti-inflammatory, anthelmintic, anticancer and antioxidant activities [8,11,12]. The traditional uses and the reported biological activities suggest that *C. orbiculata* might be a good wound-healing agent. A study by Mhlongo et al. 2022, showed that the ethyl acetate extract of *C. orbiculata* has wound-healing activity [13]. The extract, however, did not show anti-inflammatory and collagen production activities.

Wound healing is a complex and highly regulated physiological process that aims to restore the anatomical structure and function of injured tissue [14]. This process involves various growth factors and cytokines including vascular endothelial growth factor (VEGF), platelet-derived growth factor (PDGF), transforming growth factor-beta (TGF-β), fibroblast growth factor (FGF), tumor necrosis factor-alpha (TNF-α) and interleukin 1 (IL-1) [15,16,17]. Wound healing is divided into four integrated phases namely hemostasis, inflammation, proliferation, and tissue remodeling. Hemostasis, comprising vascular constriction, platelet aggregation and coagulation is intended to stop bleeding caused by the injury [17,18]. The inflammatory phase involves the infiltration of the wound site by neutrophils and monocytes to fight off any microorganisms that might have invaded the wound [18]. The proliferation phase is characterized by tissue reconstruction through the formation of granulation tissue (involving fibroblasts), angiogenesis (involving endothelial cells), wound contraction and epithelialization (involving keratinocytes) [15,18,19]. Fibroblasts and myofibroblasts cause the wound to contract by pulling the wound edges together. Epithelial cells will migrate from the wound edges, divide and proliferate along the surface of the granulation tissue until these cells form a complete sheet that covers the wound [20]. In the last phase of remodeling, there is collagen remodeling and vascular maturation, eventually leading to the flattening and strengthening of the scar [21].

Many factors can affect the wound-healing process resulting in chronic, non-healing wounds. Chronic wounds are those wounds that fail to heal in a timely and orderly manner [22]. These wounds are a silent epidemic and a global threat to the health of many individuals worldwide [23]. They affect the physical and mental health of patients, they lead to severe disability, multiple organ failure and eventually the death of the patient [24]. The discovery of effective wound healing agents to combat chronic wounds is, therefore, of paramount importance. 

We demonstrated previously that AgNPs synthesized using extracts of *C. orbiculata* (*Cotyledon*-AgNPs) have exceptional antibacterial and anti-inflammatory activities [25]. Because *C. orbiculata* is also used in traditional medicine for the treatment of wounds, we set out to investigate the wound-healing properties of extracts of *C. orbiculata* as well as *Cotyledon*-AgNPs. 

## 2. Results and Discussion

### 2.1. Phytochemical Analysis and Antioxidant Studies

The presence of phytochemicals such as polyphenols, flavanols, tannins and flavonols in *C. orbiculata* water extract and *Cotyledon*-AgNPs was investigated. As shown in Table 1, polyphenols and flavanols were shown to be 15.07 ± 1.31 mg GAE/g and 0.71 ± 0.04 mg CE/g in *Cotyledon*-AgNPs as compared to those of the extract which were 37.39 ± 0.18 mg GAE/g and 4.34 ± 0.65 mg CE/g, respectively. This may suggest that some of the polyphenols and flavanols were involved in the synthesis of the AgNPs. The synthesis of AgNPs using polyphenols has been previously reported [26,27,28]. Flavonols on the other hand were higher in *Cotyledon*-AgNPs as compared to the extract with the values of 15.64 ± 0.70 and 1.67 ± 0.16 mg QE/g, respectively. This could mean that flavonols are the main phytochemicals involved in the synthesis of *Cotyledon*-AgNPs. Flavonoids can form stable complexes by chelating metal ions through their multiple hydroxyl (–OH) groups. They are responsible for the production of nanoparticles through reduction as well as nanoparticle growth, nucleation, and stabilization [29,30]. Several studies obtained similar results where the synthesized AgNPs exhibited more flavonols than the plant extract [31,32,33]. Although the involvement of proteins, carbohydrates, terpenoids and alkaloids has been reported in metallic nanoparticle synthesis [34], polyphenols and flavonols are the major phytochemicals involved in this process [29,34]. Phytochemicals act as both the reducing and stabilizing agents in metallic nanoparticle synthesis [35]. 

Phenolic compounds, including flavonols, are not only reducing agents and metal chelators, but they are also good free radical scavengers. Due to their reducing and radical scavenger properties, phenols and flavanols have been reported to possess significant antioxidant activities. The antioxidant activities of the *C. orbiculata* water extract and *Cotyledon*-AgNPs were determined using the Ferric Reducing Antioxidant Power (FRAP) and 2′-Azino-Bis-3-Ethylbenzotiazolin-6- Sulfonic Acid (ABTS) assays. The FRAP assay directly measures the amount of antioxidants in a sample by measuring their ferric-reducing activity [36]. However, other antioxidant assays such as ABTS operate on free radical inhibition via hydrogen atom transfer [37]. As shown in Table 1, both the *C. orbiculata* water extract and the *Cotyledon*-AgNPs exhibited some antioxidant activity. In the FRAP assay, the *C. orbiculata* water extract showed more reducing activity than the *Cotyledon*-AgNPs. The *Cotyledon*-AgNPs were able to reduce approximately half the amount of ferric ions compared to the *C. orbiculata* water extract. This finding corresponds to the total polyphenol content (TPC) results, where approximately half of the polyphenols present in the extracts were used in the synthesis of the *Cotyledon*-AgNPs. Literature reports that the amount of iron reduced in the FRAP assay can be correlated with the amount of antioxidants present [38,39]. This could, therefore, mean that of all the phytochemicals present on the *Cotyledon*-AgNPs, polyphenols are the ones responsible for the ferric-reducing activity in the FRAP assay. On the other hand, the ABTS assay presented the *Cotyledon*-AgNPs as more active than the *C. orbiculata* water extract with values of 134.54 ± 20.59 and 91.14 ± 0.04, respectively. The difference in these results is indicative of the different mechanisms between the FRAP and the ABTS assays. Flavanols, phenolic acids, flavonols and tannins may be responsible for the reported bioactivities of *C. orbiculata*, as they have been shown to possess antimicrobial and anti-inflammatory activities [40]. These phytochemicals may as well be attributed to the traditional use of the plant. Increased antioxidant activity of AgNPs has been shown to increase their wound healing capabilities [41]. High ROS levels interfere with fibroblast proliferation, and oxidative stress causes damage to cell membranes, proteins and lipids resulting in delayed wound healing [41]. Therefore, antioxidant substances that can scavenge ROS and maintain non-toxic ROS levels in the wound tissues could improve healing [42]. Several biomaterials with antioxidant properties have been developed. In a study by Castro et al. 2015, an antioxidant dressing was prepared and tested using in vitro and in vivo methods. The dressing was shown to have protective effects by reducing ROS levels in fibroblasts; it also regulated the expression of inflammation related genes. In the in vivo studies, the antioxidant dressing promoted faster wound healing compared to the control [43]. 

### 2.2. Cell Viability and Growth Rate

**Figure 1:**
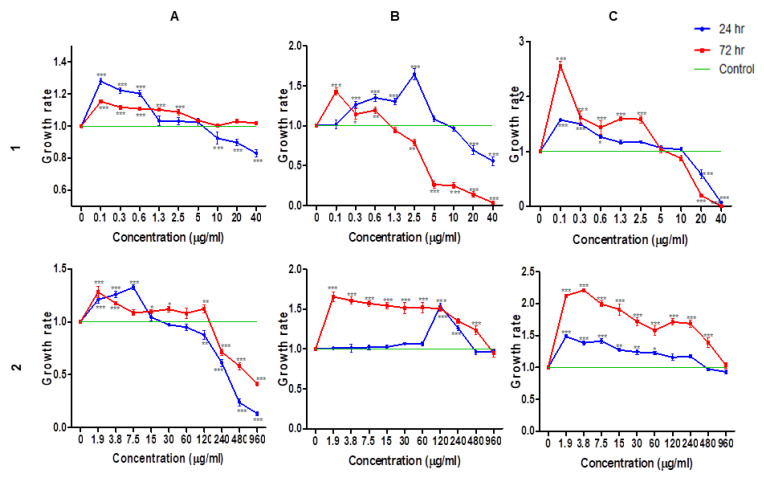
Effects of (**1**) *Cotyledon*-AgNPs and (**2**) *C. orbiculata* water extract on the cell viability and growth rate of (**A**) KMST-6, (**B**) HaCaT and (**C**) CHO cells after 24 and 72 h. Each value represents mean ± standard error of the mean (SEM); statistical significance of the *C. orbiculata* water extract and *Cotyledon*-AgNPs-treated cells when compared to the untreated cells is indicated with * for *p* < 0.05, ** *p* for <0.01, and *** *p* < 0.001.

The cell viability and growth-promoting effects of the *C. orbiculata* water extract and *Cotyledon*-AgNPs were assessed on KMST, HaCaT and CHO cells using the WST-1 assay. The viability of cells treated with the *C. orbiculata* water extract and the *Cotyledon*-AgNPs were both dose and time-dependent. The *Cotyledon*-AgNPs reduced the viability of HaCaT and CHO cells at higher concentrations (≥5 µg/mL for HaCaT and ≥20 µg/mL for CHO); however, they were non-toxic to KMST-6 cells at all the concentrations tested. These results correspond to the findings by Zanette et al. 2011, where AgNPs toxicity to HaCaT cells increased with increasing incubation time and concentration [44]. Interestingly, at lower concentrations, the *Cotyledon*-AgNPs increased the rate of cell growth (Figure 1). At concentrations between 0.1–2.5 µg/mL, *Cotyledon*-AgNPs increased the growth of KMST-6 and CHO cells at both 24 and 72 h. The *Cotyledon*-AgNPs also increased the growth rate of HaCaT cells at concentrations between 0.1 and 2.5 and 0.1 and 1.3 µg/mL at 24 and 72 h, respectively. 

The *C. orbiculata* water extract did not reduce the viability of HaCaT and CHO cells at both 24 and 72 h incubation. However, they reduced the viability of KMST-6 cells at concentrations higher than 240 µg/mL. The *C. orbiculata* water extract promoted cell growth at most of the concentrations tested especially the lower concentrations. Unlike the *Cotyledon*-AgNPs which decreased cell growth as incubation time increased, the *C. orbiculata* water extract actually increased the growth of cells with increasing incubation time. The concentrations of 2.5 µg/mL (*Cotyledon*-AgNPs) and 15 µg/mL (*C. orbiculata* water extract) were used for the scratch wound healing assay as these showed a stable trend amongst the different cell lines. 

### 2.3. Wound Healing Activity

**Figure 2:**
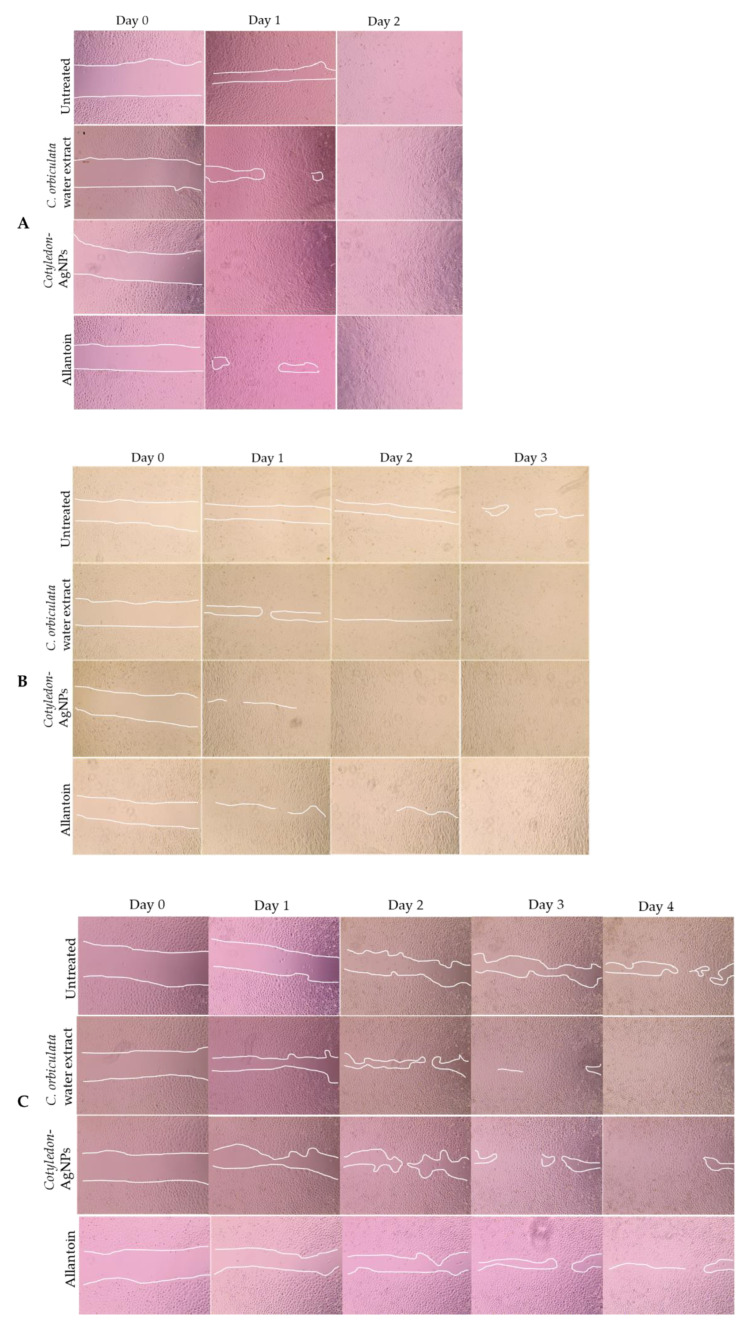
Scratch assay analysis of *Cotyledon*-AgNPs (2.5 µg/mL) and *C. orbiculata* water extract (15 µg/mL) on HaCaT (**A**), CHO (**B**) and KMST-6 (**C**) cells. The white lines in the images are boundaries of the scratched gap.

**Figure 3:**
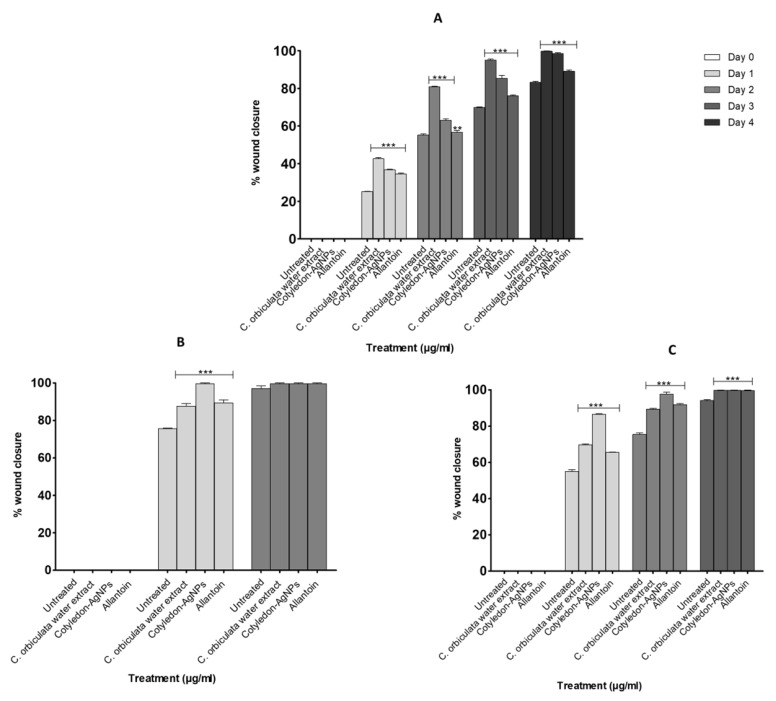
Percentage wound closure rates of *Cotyledon*-AgNPs and *C. orbiculata* water extract-treated KMST-6 (**A**), HaCaT (**B**) and CHO (**C**) cells. Each value represents mean ± standard error of the mean (SEM); statistical significance was indicated with ** *p* for < 0.01 and *** *p* < 0.001.

The wound healing activity of the *C. orbiculata* water extracts and *Cotyledon*-AgNPs was tested using the scratch assay. This is a popular, simple method used to determine the wound-healing activities of different compounds [45]. It is related to the proliferation phase of wound healing which involves the migration and proliferation of fibroblasts and keratinocytes. This assay mimics the in vivo migration of cells during wound healing [46]. Both the *C. orbiculata* water extracts and the *Cotyledon*-AgNPs were able to close the scratched gap faster than the Negative Control (untreated cells) in all cell lines (Figure 2). The images show a time-dependent increase in the density of cells in the scratched area until the gap closes. This strongly indicates that the *C. orbiculata* water extracts and the *Cotyledon*-AgNPs possess both cell migration and proliferation properties. Therefore, it can be concluded that *C. orbiculata* promotes wound healing activities via increased migration and proliferation of fibroblasts, keratinocytes and epithelial cells. Our findings support literature that reports that AgNPs promote wound healing through the differentiation of myofibroblasts to fibroblasts, wound contractility and epidermal re-epithelialization through keratinocyte proliferation and migration [2,47]. Similar observations where plant-derived AgNPs promoted wound healing via migration of fibroblasts [48,49] and keratinocytes [50] have been previously reported. In HaCaT and CHO cells, *Cotyledon*-AgNPs (at 2.5 µg/mL) exhibited more activity than the water extract, in-fact at this concentration, *Cotyledon*-AgNPs had more activity than the Positive Control (treatment with 15 µg/mL allantoin) (Figure 2 and Figure 3). Chinnasamy et al. 2019 reported similar results with *Melia azedarach* synthesized AgNPs which exhibited increased wound healing activities compared to the *M. azedarach* extract alone [51]. This might be due to the nanoparticles’ small size which increases their surface area to volume ratio thus increasing their activity [2]. It might also be due to phytochemicals present on the nanoparticles (such as polyphenols and flavonols as shown in Table 1) which can increase their bioavailability and enhance their activity [52]. In KMST-6 cells, however, the water extract showed more activity than the *Cotyledon*-AgNPs. However, considering that a lower concentration of the AgNPs (2.5 µg/mL) was used; the latter statement cannot be confirmed. Although *Cotyledon*-AgNPs were more active than the *C. orbiculata* water extract, the activity of the extract was also significant as it showed good activity at very low concentrations (≤15 µg/mL). These findings show that *C. orbiculata* most likely contains compounds with highly active wound-healing properties, and therefore, provide some scientific evidence supporting its traditional use. Other medicinal plants were also recorded to have good wound healing activity, these include *Plantago australis* [53], *Spirulina platensis* [54], *Alternanthera sessilis* [55], *Commiphora molmol*, *Aloe vera* [56] and *Achillea eriophora* [57].

Figure 2 and Figure 3 show that all cell lines exhibited different migration rates. It can be noted that complete gap closure of HaCaT, CHO and KMST-6 cells took 48, 72 and 96 h, respectively (Figure 3). The daily percentage wound closure rates were determined using the equation stated in Section 3.5.3, these rates correspond to the findings from the scratch assay (Figure 2). After exposure to the *C. orbiculata* water extract and the *Cotyledon*-AgNPs, HaCaT and CHO cells responded in a similar pattern, while the response of KMST-6 cells was different. In these cells, the wound closure rates were increased in the first 24 h compared to KMST-6 cells which had a more evenly spread cell migration pattern. The results in Figure 3 where almost complete wound closure was observed in HaCaT cells after day 1 corresponds to the increased growth rate in *Cotyledon*-AgNPs (2.5 µg/mL) treated HaCaT cells after 24 h as shown in Figure 1. 

### 2.4. Gene Expression Studies

**Figure 4:**
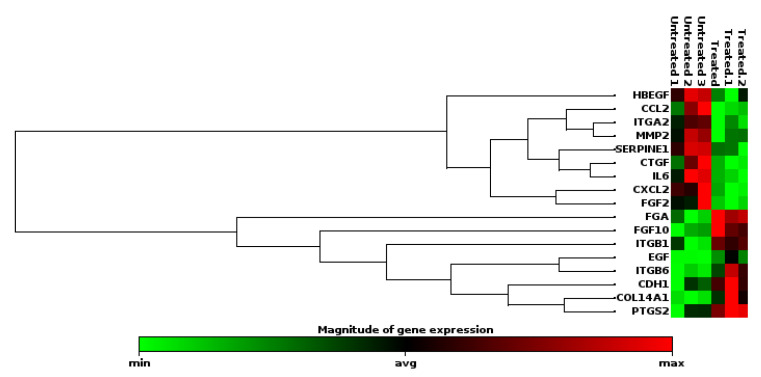
Clustergram of DEGs expressed in *Cotyledon*-AgNPs treated KMST-6 cells.

**Figure 5:**
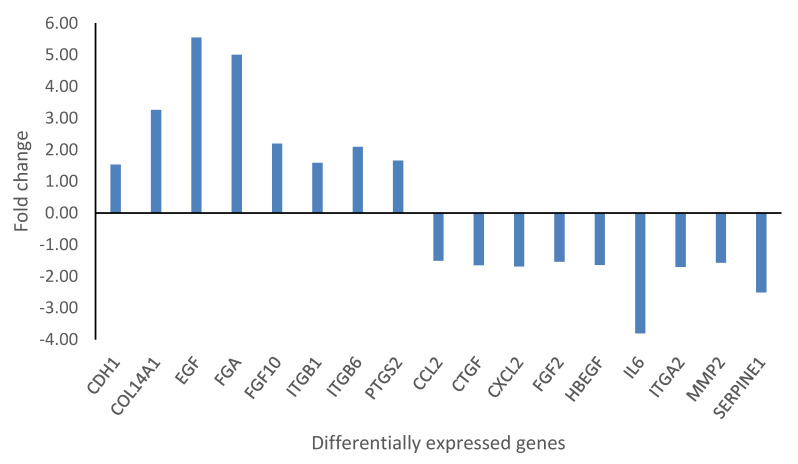
Fold changes in the DEGs expressed after KMST-6 cells were treated with *Cotyledon*-AgNPs. Genes with a fold change of ≥±1.5 and p-values of <0.05 were considered DEGs.

**Figure 6:**
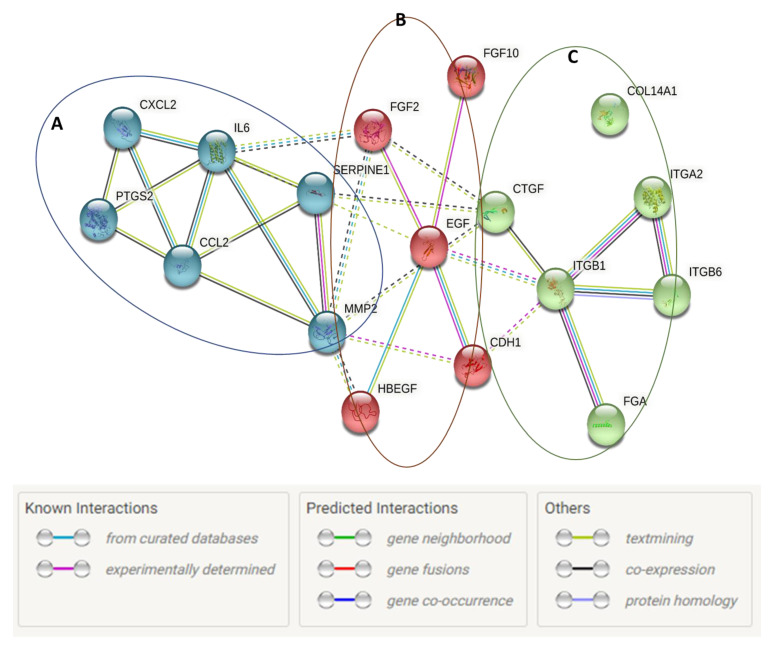
Protein networks showing the interactions between DEGs in the *Cotyledon*-AgNPs treated KMST-6 cells. These networks were determined using the STRING database.

KMST-6 (fibroblasts) cells were used for gene expression studies. This is because fibroblasts are critical in all stages of wound healing and are involved in key processes such as extracellular matrix production, collagen deposition and wound contraction [58,59]. Gene expression studies investigating the expression levels of 86 wound healing-related genes were conducted on KMST-6 cells. In *Cotyledon*-AgNPs treated cells, only 17 of the 86 genes were differentially expressed (Figure 4). Eight (CDH1, COL14A1, EGF, FGA, FGF10, ITGB1, ITGB6, PTGS2) of the differentially expressed genes (DEGs) were upregulated while the other nine (CCL2, CTGF, CXCL2, FGF2, HBEGF, IL6, ITGA2, MMP2, SERPINE1) were downregulated (Figure 4 and Figure 5). Genes with a fold change of ≥±1.5 and *p*-values of <0.05 were considered DEGs. DAVID and STRING analysis clustered DEGs into three different groups (Figure 6). Cluster A consisted of five downregulated genes (CCL2, CXCL2, IL6, MMP2, SERPINE1) and one upregulated gene (PTGS2). These five genes are involved in the inflammatory phase of wound healing. Cluster A genes promote inflammation, mostly via leucocyte chemotaxis, cytokine production and prostaglandin production [60,61,62]. Although inflammation plays an important role during the initial stages of wound healing, it is known that extended inflammation can lead to chronic wounds [63]. Based on the gene expression study which shows that most of the genes involved in inflammation are downregulated it can be concluded that the *Cotyledon*-AgNPs have anti-inflammatory activity. This is in agreement with our previous study which demonstrated the anti-inflammatory activity of *Cotyledon*-AgNPs in THP-1 macrophages [25]. 

As shown in Table 2, the downregulated genes function in a number of pathways, including TNF signaling and cytokine production, all of which lead to inflammation. 

Cluster B contained three (CDH1, EGF, FGF10) upregulated and two (FGF-2, HB-EGF) downregulated genes. Genes in this cluster are mainly involved in epithelial cell proliferation and migration. The upregulated genes stimulate keratinocyte proliferation and migration and also increase the tensile strength of the new skin [64,65,66]. These genes also improve collagen construction and stimulate the formation of the extracellular matrix (ECM) [64,67]. It is possible that the downregulated genes are downstream of the upregulated ones and may, therefore, be activated with increased exposure to the *Cotyledon*-AgNPs. Although the downregulated genes promote keratinocyte proliferation and migration, they also promote inflammation [68,69]. This might be the reason why they were downregulated. These genes modulate inflammatory cell recruitment and activation, particularly macrophages and fibroblasts [69,70]. Upon activation, fibroblasts produce and secrete pro-inflammatory cytokines (IL6, IL-1B, IL8), chemokines (CCL2) and prostanoids (prostaglandin E2) [71,72,73].

Cluster B genes highly correlate with those in cluster C, these genes function using the same pathways (PI3K-Akt signaling pathway, Regulation of actin cytoskeleton, Rap1 signaling pathway and Focal adhesion) as shown in Table 2. Group C genes are involved in cellular adhesion which regulates cell differentiation and migration [74]. Cell adhesion is the ability of cells to stick to each other or to the ECM through molecules such as collagen, fibronectin, and laminin [75]. It is essential in cell communication and regulation as well as in the development and maintenance of tissues. Cell adhesion is also essential in wound healing as it stimulates signals that regulate important wound-healing processes such as cell differentiation, migration and survival [74]. Integrins are the main mediators of cell attachment to the ECM. They form cellular receptors for the extracellular environment and adjacent cells [76]. Their binding to extracellular ligands such as fibronectin and laminin promotes intracellular signaling which eventually causes cell migration [77]. The upregulated integrin genes ITGB1, ITGB6 and FGA are not only essential in cell adhesion but also in angiogenesis. FGA leads to the formation of an insoluble fibrin matrix. The major function of fibrin is its involvement in the formation of blood clots, therefore, reducing blood loss. Fibrin also forms the temporary ECM in the wounded area and plays important roles in tissue repair and cell migration during angiogenesis [78]. 

**Figure 7:**
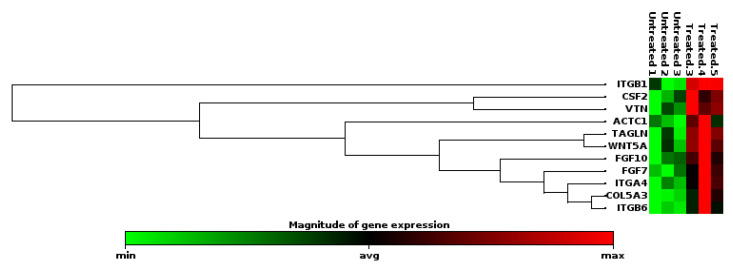
Clustergram of DEGs expressed in *C. orbiculata* water extract-treated KMST-6 cells.

**Figure 8:**
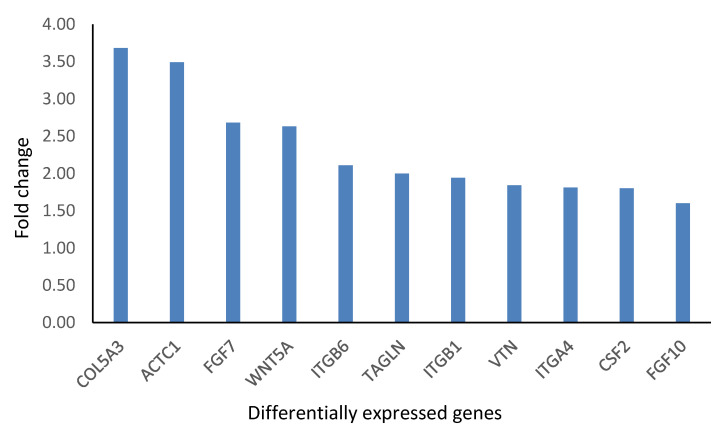
Fold changes in the DEGs expressed after KMST-6 cells were treated with *C. orbiculata* water extract. Genes with a fold change of ≥±1.5 and p-values of <0.05 were considered as DEGs.

**Figure 9:**
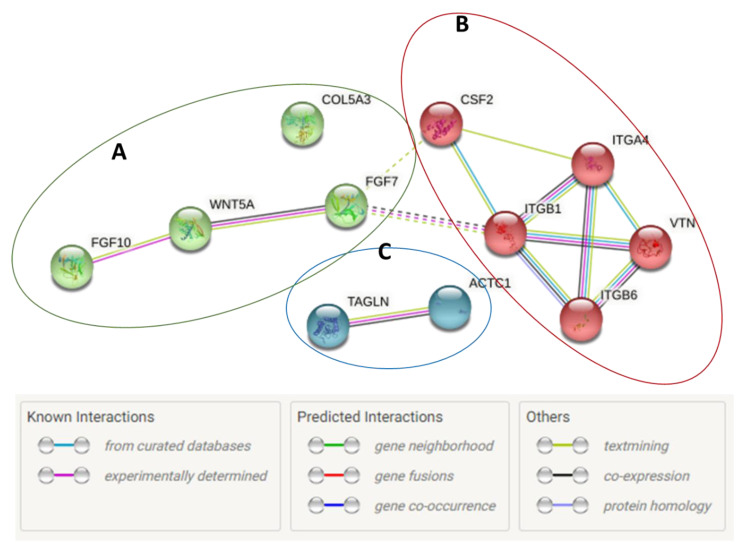
Protein networks showing the interactions between DEGs expressed in KMST-6 cells treated with *C. orbiculata* water extract. These networks were determined using the STRING database.

The effects of the *C. orbiculata* water extract on wound healing genes were also determined. Of the 86 genes tested, only 11 (COL5A3, ACTC1, FGF7, WNT5A, ITGB6, TAGLN, ITGB1, VTN, ITGA4, CSF2, FGF10) were differentially expressed; all of them were upregulated (Figure 7 and Figure 8). Two (FGF10, ITGB1) of these genes were also upregulated in *Cotyledon*-AgNPs treated cells (Figure 5), both genes are involved in the proliferation and migration of epithelial cells. Both STRING and the DAVID software were used to analyze these results. These upregulated genes are separated into three clusters all of which promote cell proliferation and migration (Figure 9). Cluster A genes (particularly FGF10 and WNT5A) are involved in epithelial cell proliferation and migration especially fibroblasts and keratinocytes. The WNT protein family is made up of glycoproteins that regulate cell proliferation, in this family WNT5A was discovered as a key regulator of fibroblast proliferation [79,80].

Cluster B genes are involved in cell adhesion mostly by integrin, leading to increased cell survival. Cluster C consists of two genes (ACTA1 and TAGLN) which are involved in cytoskeleton organization and cell motility [81]. Actin is a structural protein that makes up the cell cytoskeleton while TAGLN is an actin crosslinking protein [81,82]. These genes ultimately support cell differentiation, proliferation, and migration [83]. Genes differentially expressed in response to treatment with *C. orbiculata* water extract were in similar categories to those expressed by the *Cotyledon*-AgNPs (cluster B, FGF10 and cluster C, ITGB1), they function using similar pathways as shown in Table 2 and Table 3. This shows that the *C. orbiculata* water extract and the *Cotyledon*-AgNPs promote wound healing using similar mechanisms which include cell proliferation and migration. This can confirm the results in Table 1 which show that the same phytochemicals present in the extract and responsible for the bioactivities are also present on the nanoparticles. Results obtained using the WST-1 assay (Figure 1) and the scratch assay (Figure 2) support this finding. 

## 3. Materials and Methods

### 3.1. Materials

The Folin–Ciocalteu reagent, Sodium carbonate, 4-Dimethylamino-cinnamaldehyde, Methanol, Hydrochloric acid, Acetate buffer, 2, 4, 6-tripyridyl-S-triazine, Ascorbic acid, ABTS radical, WST-1 reagent, Hams-F12 medium, Dulbecco’s modified eagle medium (DMEM), Phosphate-buffered saline (PBS), Fetal bovine serum (FBS), Pen-strep (penicillin and streptomycin), were obtained from Sigma-Aldrich (St. Louis, Mo, USA). Gene expressions kits (RNeasy Mini Kit, RT2 First Strand Kit, Human wound healing RT2 Profiler PCR Array) were from Qiagen (Hilden, Germany).

### 3.2. Plant Extract Preparation and Synthesis of Cotyledon-AgNPs

The plant extracts and *Cotyledon-*AgNPs used in this study were prepared as described previously [25]. 

### 3.3. Phytochemical Analysis

#### 3.3.1. TPC

The TPC of the *C. orbiculata* water extract and the *Cotyledon*-AgNPs was determined using the Folin–Ciocalteu method [84]. In a 96-well plate, 25 µL of the samples and standards were mixed with 125 µL of the Folin–Ciocalteu reagent (10%). After 5 min, 100 µL of sodium carbonate was added to the mixture. The plates were incubated for 2 h at room temperature and then read using a spectrophotometer at an absorbance of 765 nm. 

#### 3.3.2. Total Flavonoid Content (TFC)

##### The Flavanol Content

The flavanol content was determined using the method by [85] with modifications. In short, 50 µL of the *C. orbiculata* extract and *Cotyledon*-AgNPs were mixed with 250 µL of 4-Dimethylamino-cinnamaldehyde. The mixture was incubated for 30 min at room temperature after which the absorbance was read at 640 nm. Results were expressed as milligram catechin equivalent per gram (mg CE/g).

##### The Flavonol Content

The flavonol content was also determined according to the method in [85], with modifications. Briefly, 12.5 µL of 0.1% HCl in methanol and 225 µL of 2% HCl solutions were added to 12.5 µL of the *C. orbiculata* extract and *Cotyledon*-AgNPs. The mixture was incubated for 30 min at room temperature and the absorbance was read at 360 nm. Results were expressed as milligram quercetin equivalent per gram (mg QE/g). 

### 3.4. Antioxidant Studies

The antioxidant activity of the *C. orbiculata* water extract and the *Cotyledon*-AgNPs was determined using the most common colorimetric assays namely FRAP and ABTS. 

#### 3.4.1. FRAP

This method is based on the ability of antioxidants to reduce the ferric ion (Fe^3+^) to ferrous ion (Fe^2+^). In the presence of antioxidants at low pH, the ferric tripyridyl triazine complex (colorless) is reduced to its ferrous form which is a blue-colored complex. The ferric-reducing power of the *C. orbiculata* water extract and *Cotyledon*-AgNPs was evaluated according to the method developed by Benzi and Strain in 1996 [86]. The FRAP reagent was prepared by mixing 10 mL of acetate buffer (300 mM), 1 mL of 2, 4, 6-tripyridyl-S-triazine (TPTZ) (10 mM) and 1 mL of FeCl_3_·6H_2_O (20mM). In a 96-well plate, the FRAP solution (300 μL) was mixed with 10 μL of the samples (*C. orbiculata* water extract and *Cotyledon*-AgNPs) and incubated at 37 °C for 30 min. A spectrophotometer was used to measure the absorbance readings at 593 nm. Ascorbic acid was used in the preparation of the standard solutions. The absorbance of the samples was compared to a standard curve and the values were expressed as µmol AAE/g.

#### 3.4.2. ABTS

The ABTS assay is used to measure the total antioxidant activity of substances. It is based on the ability of antioxidants to scavenge the ABTS (2,2′-azinobis-(3-ethylbenzothiazoline-6-sulfonic acid)) radical cation relative to the Trolox standard. This assay was performed according to the method used by Dube et al. 2017 [84]. Briefly, the ABTS radical mixture was prepared by mixing 5 mL ABTS (7 mM) and 88 μL potassium peroxodisulfate (K_2_S_2_O_8_) (140 mM). The mixture was incubated in the dark for 24 h at room temperature and was diluted with ethanol to read an absorbance of approximately 2.0 (±0.1) at 734 nm, as the control; 300 μL of the diluted ABTS radical mixture was added to 25 μL of the *C. orbiculata* extracts, *Cotyledon*-AgNPs and standard in 96 well plates. The plates were incubated for 30 min at room temperature and absorbance readings were taken at 734 nm. The results were expressed as µmol TE/g.

### 3.5. Cell Culture Studies

#### 3.5.1. Cell Culture

HaCaT, KMST-6 and CHO cells were obtained from the DSI/Mintek NIC (Nanotechnology Innovation Centre) laboratory at the University of the Western Cape (South Africa). The HaCaT and KMST-6 cells were cultured in Dulbecco’s modified eagle medium (DMEM) supplemented with 10% Fetal bovine serum (FBS) and 1% Penicillin/Streptomycin. The CHO cells were grown in a Hams-F12 medium containing 10% FBS and 1% Penicillin/Streptomycin. All the cells were maintained in a humidified atmosphere of 5% CO_2_ in a 37 °C incubator (SL SHEL LAB, Sheldon manufacturing, Cornelius, OR, USA). 

#### 3.5.2. Cell Viability and Growth Rate

Both cell viability and growth rate were determined using the WST-1 (Sigma-Aldrich, St. Louis, MO, USA) assay according to the method by [87]. Cells were seeded in 96 well plates at a density of 1 × 10^5^ cell/mL and incubated for 24 h. The growth media was replaced with a medium containing *C. orbiculata* water extract and *Cotyledon*-AgNPs at different concentrations and the cells were further incubated for 24 to 72 h. After incubation, the conditioned media was replaced with 10% WST-1 reagent (diluted in respective medium) and then plates were incubated for an additional 3 h. The absorbance was measured at 440 nm (reference 630 nm) using a microplate reader (POLARstar Omega plate reader, BMG-Labtech, Ortenberg, Germany). Cell viability was expressed as a percentage of the absorbance of treated cells to control (untreated) cells. The cell growth rate was determined using cell viability; 100% cell viability (untreated cells) was used as the control for growth rate with a value of 1. 

#### 3.5.3. Scratch Assay

The scratch assay was determined using a method by [55], with modifications. HaCaT, KMST-6 and CHO cells were seeded in 24 well plates at a density of 2 × 10^5^ cell/mL and were incubated for 24 h at 37 °C in 5% CO_2_. After 24 h, a monolayer of confluent cells was observed. To create a scratch, the cell monolayer was scraped in a straight line using a sterile pipette tip. Cellular debris was removed by washing the wells with fresh medium. The cells were incubated at 37 °C in the presence of *C. orbiculata* water extract and the *Cotyledon*-AgNPs. The concentrations of *C. orbiculata* water extract and the *Cotyledon*-AgNPs were 15 and 2.5 µg/mL, respectively. Allantoin (15 µg/mL) which is a plant-derived commercial drug and known to induce cell growth was used as a positive control [55]. Untreated scratched cells were used as the negative control. All treatments and controls were placed in a medium containing 1% FBS. The assay was conducted in triplicate. Wound closure was monitored at different time intervals using a digital light microscope (EVOS XL Core Cell Imaging System, CA, USA). Images were analyzed using the Image J software. The percentage of wound closure was obtained using the equation,

$$\mathrm{Wound}\;\mathrm{closure}\;\left(\%\right)=\frac{\mathrm{Wound}\;\mathrm{area}\;\left(0\;h\right)-\;\mathrm{Wound}\;\mathrm{area}\;\left(t\;h\right)\;}{\mathrm{Wound}\;\mathrm{area}\;\left(0\;h\right)}\times 100$$

### 3.6. Gene Expression Studies

KMST-6 cells were seeded at a density of 2 × 10^5^ cell/mL in a 25 cm^2^ cell culture flask, the flasks were treated with *C. orbiculata* water extracts (15 µg/mL) and *Cotyledon*-AgNPs (2.5 µg/mL). Untreated flasks were used as the control. This was conducted in triplicate. The cells were collected into Eppendorf tubes and prepared for RNA extraction. Total RNA was extracted from the untreated and treated cells using the RNeasy Mini Kit (Qiagen, Hilden, Germany) according to the manufacturer’s protocol. The concentration of the RNA was determined using a Qubit^®^ 2.0 Fluorometer (Invitrogen by Life Technologies, Carlsbad, CA, USA) according to the manufacturer’s protocol. cDNA was synthesized from RNA using the RT2 First Strand Kit (Qiagen, Hilden, Germany) following the manufacturer’s protocol. The produced cDNA was used as a template for RT-qPCR which was conducted using the Human wound healing RT2 Profiler PCR Array (Qiagen, Hilden, Germany) following the manufacturer’s instructions. The experiment was carried out on the Roche LightCycler 480 (Roche, Basel, Switzerland) in the department of the Institute of Microbial Biotechnology and Metagenomics (IMBM) at the University of the Western Cape. Data collected from the LightCycler 480 was analyzed at GeneGlobe Data Analysis Center under Qiagen. 

The relative changes in gene expression were calculated using cycle threshold (CT) values. The CT values were first normalized to those of used housekeeping genes. The fold change was then calculated using the 2^−ΔΔCT^formula adapted from Livak and Schmittgen [88]. Genes with a fold change of ≥±1.5 and p-values of <0.05 were considered as DEGs and were used for further analysis. The Database for Annotation, Visualization and Integrated Discovery (DAVID; version 6.7) and Search Tool for the Retrieval of Interaction Genes/Proteins (STRING; https://string-db.org/, accessed on 5 November 2022) pathways were used to further analyze the different interactions of the DEGs. 

### 3.7. Statistical Analysis

Statistical analysis of the data was conducted using the GraphPad Prism 6 software. All assays were performed in triplicates and the results were expressed as mean ± standard error of the mean (SEM). Statistical significance was indicated as * for *p* < 0.05, ** *p* for < 0.01, *** *p* < 0.001 and **** for *p* < 0.0001.

## 4. Conclusions

It can be concluded that the medicinal plant, *C. orbiculata* possesses antioxidant and wound-healing activities. Both the *C. orbiculata* water extract and the *Cotyledon*-AgNPs showed good antioxidant activities which can increase their wound healing capabilities. They both increased the growth of HaCaT, KMST-6 and CHO cells at low concentrations and promoted cell migration in these cells in the scratch assay. The activity of the *Cotyledon*-AgNPs can be attributed to their small size which increases their surface area to volume ratio, hence increasing their activity. It might also be due to phytochemicals present on the nanoparticles which can increase their bioavailability and enhance their activity. Gene expression studies confirmed that the *C. orbiculata* water extract and *Cotyledon*-AgNPs promote wound healing through the activation of genes involved in the proliferation phase of wound healing. *C. orbiculata* extract and *Cotyledon*-AgNPs promote keratinocyte and fibroblast proliferation and migration by upregulating genes such as FGF7 and FGF10. *Cotyledon*-AgNPs are also involved in hemostasis which promotes clotting thus reducing blood loss. While *C. orbiculata* has been used in traditional medicine to treat skin conditions and wounds, this study scientifically shows that this plant contains wound-healing properties. We previously demonstrated that *Cotyledon*-AgNPs have significant antibacterial activity against microorganisms that commonly infect wounds. We also showed previously that these nanoparticles have anti-inflammatory activity. The current study shows that the *Cotyledon*-AgNPs also promote the growth of keratinocytes and fibroblasts at specific concentrations. We have also shown that these nanoparticles are not toxic at these concentrations. Taken together these findings demonstrate that *Cotyledon*-AgNPs have immense potential for application as novel wound healing agents.

## Figures and Tables

**Table 1:** Phytochemical analysis and antioxidant studies.

Treatment (1 mg/mL)	*C. orbiculata* Water Extract	*Cotyledon*-AgNPs
Polyphenols (mg GAE/g)	37.39 ± 0.18	15.07 ± 1.31
Flavonols (mg QE/g)	1.67 ± 0.16	15.64 ± 0.70
Flavanol and tannins (mg CE/g)	4.34 ± 0.65	0.71 ± 0.04
FRAP µmol AAE/g	258.84 ± 2.75	127.28 ± 10.9
ABTS µmol TE/g	91.14 ± 0.04	134.54 ± 20.59

**Table 2:** Enriched pathways in which the *Cotyledon*-AgNPs expressed DEGs function through.

Pathways	Involved Genes	*p*-Value
PI3K-Akt signalling pathway	EGF, FGF10, FGF2, ITGB1, ITGB6, ITGA2, IL6	7.9 × 10^−5^
Regulation of actin cytoskeleton	EGF, FGF10, FGF2, ITGB1, ITGB6, ITGA2	8.4 × 10^−5^
Rap1 signalling pathway	EGF, FGF10, FGF2, ITGB1, CHD1	1.2 × 10^−3^
Focal adhesion	ITGA2, ITGB1, ITGB6, EGF	1.1 × 10^−2^
ECM-receptor interaction	ITGA2, ITGB1, ITGB6	1.7 × 10^−2^
IL-17 signalling pathway	CXCL2, CCL2, IL6, PTGS2	4.5 × 10^−5^
TNF signaling pathway	CXCL2, CCL2, IL6, PTGS2	1.8 × 10^−3^
NOD-like receptor signaling pathway	CXCL2, CCL2, IL6	7.3 × 10^−3^
Cytokine-cytokine receptor interaction	CXCL2, CCL2, IL6	1.6 × 10^−2^

**Table 3:** Enriched pathways in which *C. orbiculata* water extract expressed DEGs function through.

Pathways	Involved Genes	*p*-Value
PI3K-Akt signalling pathway	FGF10, FGF7, COL5A3, ITGB1, ITGB6, ITGA4, VTN	1.1 × 10^−6^
Regulation of actin cytoskeleton	FGF10, FGF7, ITGB1, ITGB6, ITGA4	9.4 × 10^−5^
ECM-receptor interaction	ITGB1, ITGB6, ITGA4, COL5A3, VTN	2.9 × 10^−6^
Focal adhesion	ITGB1, ITGB6, ITGA4, COL5A3, VTN	8.7 × 10^−5^
Rap1 signalling pathway	FGF10, FGF7, ITGB1	2.9 × 10^−2^

## Data Availability

Supporting data presented in this study are available on request from the corresponding author.
